# Classification of Time Series Gene Expression in Clinical Studies via Integration of Biological Network

**DOI:** 10.1371/journal.pone.0058383

**Published:** 2013-03-13

**Authors:** Liwei Qian, Haoran Zheng, Hong Zhou, Ruibin Qin, Jinlong Li

**Affiliations:** 1 School of Computer Science and Technology, University of Science and Technology of China, Hefei, People's Republic of China; 2 Anhui Key Laboratory of Software Engineering in Computing and Communication, University of Science and Technology of China, Hefei, People's Republic of China; 3 Department of Systems Biology, University of Science and Technology of China, Hefei, People's Republic of China; Chinese Academy of Sciences, China

## Abstract

The increasing availability of time series expression datasets, although promising, raises a number of new computational challenges. Accordingly, the development of suitable classification methods to make reliable and sound predictions is becoming a pressing issue. We propose, here, a new method to classify time series gene expression via integration of biological networks. We evaluated our approach on 2 different datasets and showed that the use of a hidden Markov model/Gaussian mixture models hybrid explores the time-dependence of the expression data, thereby leading to better prediction results. We demonstrated that the biclustering procedure identifies function-related genes as a whole, giving rise to high accordance in prognosis prediction across independent time series datasets. In addition, we showed that integration of biological networks into our method significantly improves prediction performance. Moreover, we compared our approach with several state-of–the-art algorithms and found that our method outperformed previous approaches with regard to various criteria. Finally, our approach achieved better prediction results on early-stage data, implying the potential of our method for practical prediction.

## Introduction

In the last decade, the development of a variety of techniques, such as microarray-based techniques, has enabled instant measurement of the expression of up to thousands of genes. The use of gene expression profiling allows clinical diagnosis to be made on a molecular level, thereby facilitating the choice of treatment based on the patients' genetic traits [1]. Some of these methods have been commercialized, creating tools for expression-based diagnosis and treatment prognosis [2], [3].

In the past few years, gene expression experiments have been limited to static analysis [4], [5], in which only a snapshot of gene expression for a set of samples is available. Recently, many research groups have proposed the combination of static gene expression measurements with biological networks, such as protein-protein interaction and metabolic networks, and have verified that integrative approaches can achieve better classification results than approaches that analyze only static gene expression data [6], [7], [8], [9], [10], [11].

Although static analyses are appropriate for many cases, they are less appropriate for longer-term follow-up [12]. In the case of transplant patients, for example, physicians need to determine whether or when patients' bodies start rejecting the new organ, in order to determine whether treatment with immunosuppressant drugs is required. In this scenario, classification can be improved if one takes into account not only the current state of the patient but also their past state and the changes that have occurred over time. Accounting for the temporal dynamics of gene expression affords a novel perspective on clinical studies, such as drug response prediction. Although time series analysis is promising, it also raises a number of new computational challenges. A unique challenge for clinical time series expression classification is to take into account the patient-specific rate of disease development or treatment response [13]. Another challenge is that clinical data usually have very peculiar characteristics: typically, only a small number of samples (patients) are available, each containing a large number of features (genes) [14]. As a result, in order to make reliable and sound predictions, careful development of suitable classification methods becomes a pressing issue.

Over the past several years, a few methods for classifying time series expression data have been reported, including an exhaustive search strategy to identify genes for a Bayesian classifier [14], an SVM method based on dynamic systems kernels [15], a classifier based on hidden Markov models and discriminative learning [12], a series of biclustering-based classification algorithms [16], and a tensor decomposition method to classify time series expression data [17]. One major problem of these approaches is that they merely analyze genome-wide expression profiles, which contain inherent measurement noise. Heterogeneity across samples further aggravates this problem. Moreover, gene markers selected by these approaches may be function-related and hence contain redundant information. Collectively, these factors lead to the degradation of the overall classification performance [8], [10].

Motivated by limitations of current classification methods in time series data and the good performance of network integration approaches in static gene expression, our aim in this study was to classify time series gene expression via integration of biological networks. To our knowledge, this study is the first attempt at integration of biological networks for classification of time series data. We emphasize that integration approaches in static analysis cannot be directly applied to time series data. In order to reduce the effect of the measurement noise, we have introduced a hybrid model of hidden Markov model/Gaussian mixture model (HMM/GMM) into our approach, converting the original gene expression value, which contains noise, into a discrete gene state that represents the qualitative assessment of the gene expression level. The HMM/GMM hybrid model also takes into consideration time-dependence, which is a special property of time series data. Instead of using single gene markers that may be function-related, we regard genes that show similar expression pattern as a whole (bicluster) with the hypothesis that these genes may share a common biological function. Because genes sharing an expression pattern are likely, but not certain, to be function-related, we integrated biological networks into our approach, weighting a bicluster according to its connected genes in the network. The more closely connected the genes, the more likely the genes share a common biological function, and the higher the weight. Every sample (patient) is denoted as a point in the bicluster space. Similarity between samples (distance between points in the space) can be calculated as the weighted sum of bicluster similarity. We classified a sample based on its similarity to other samples.

Here, we investigated the classification of Multiple Sclerosis (MS) patients with respect to their response to interferon beta (IFN-β) treatment. MS is one of the most prevalent autoimmune disorders, and treatment with IFN-β is widely applied to reduce the intensity and frequency of symptoms. Nevertheless, almost 50% of patients do not respond to the therapy [15]. To exacerbate matters, IFN-β therapy has been associated with a number of adverse reactions, including flu-like symptoms, transient laboratory abnormalities, menstrual disorders, increased spasticity, and dermal reactions [16]. Accordingly, it is of great importance to accurately predict patients' response to therapy ahead of, or at an early stage in treatment. In this paper, we evaluated our approach on 2 different MS datasets and compared the results of our GMM/HMM model with other discretization methods. We investigated the contribution of integrating networks within our approach and compared our method with several state-of-the-art algorithms with respect to various performance criteria. Finally, we demonstrated the potential of our approach in practical prediction.

## Materials and Methods

**Figure 1:**
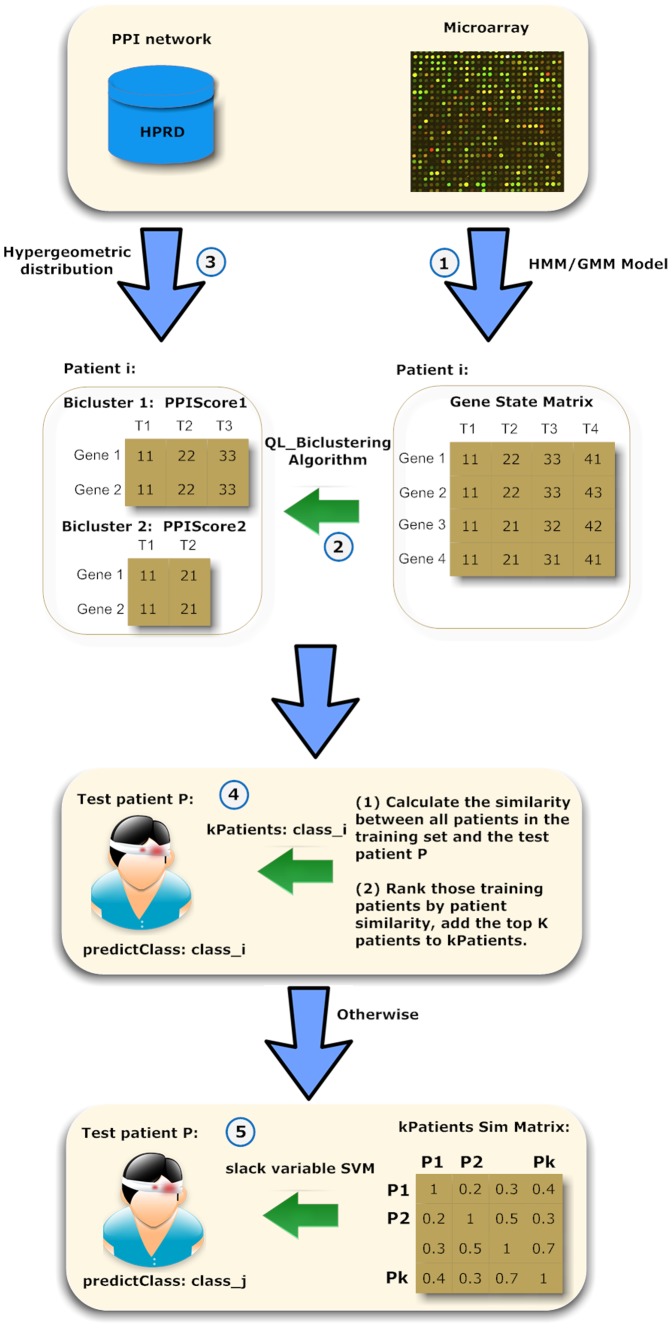
Schematic overview of classification of time series gene expression. The prediction process primarily consists of 4 or 5 steps. Firstly, gene states are inferred by an HMM/GMM hybrid model. Secondly, the QL-biclustering algorithm extracts biclusters of every patient from the gene state matrix. Thirdly, every bicluster is scored according to its genes' connection in the protein-protein interaction (PPI) network. Finally, the label of every test patient is predicted by PPI-SVM-KNN based on patient similarity, taking into account both bicluster similarity and its PPIScore.

The prediction process primarily consists of 4 steps (Figure 1). Firstly, gene states are inferred by an HMM/GMM hybrid model. Secondly, the QL-biclustering algorithm extracts biclusters of every patient from the gene state matrix. Thirdly, every bicluster is scored according to its genes' connection in the protein-protein interaction (PPI) network. Finally, the label of every test patient is predicted by PPI-SVM-KNN based on patient similarity, taking into account both bicluster similarity and its score. The software is available at http://home.ustc.edu.cn/~lwqian/PPI-SVM-KNN/PPI.html.

### 1. Dataset

#### 1.1 Time series gene expression dataset

We tested our approach on 2 different sets of time series microarray expression data from Baranzini and Goertsches [17], [18] to predict whether MS patients [19] will respond positively to treatment with recombinant human IFN-β (rIFN-β). The Baranzini dataset contains time expression profiles of 52 multiple sclerosis patients, of which 33 responded well, and 19 responded poorly to rIFN-β. Expression profiles of 70 genes were measured up to 7 times per patient. The first 5 observations were at a regular intervals of 3 months apart, whereas the last 2 observations were spaced 6 months apart. Seventeen patients missed a test, and hence, we have only 6 measurements. Eight patients missed 2 tests, and hence, we have only 5 measurements. All missing values are filled with a weighted mean of the 3 closest neighboring values after data normalization. The Goertsches dataset contains 25 patients, out of which 15 responded well and 10 responded poorly to rIFN-β. Expression profiles of genes were measured 5 times per patient. Here, we used the expression values of 58 genes that were also measured in Baranzini dataset. It should be mentioned here that Baranzini dataset is based on one-step kinetic reverse-transcription PCR experiments. The Goertsches dataset is based on Affymetrix DNA microarrays.

#### 1.2 PPI network

The protein-protein interaction network obtained from a public human PPI database (HPRD) [20] was used here, excluding those PPIs not related to the genes of MS dataset. After excluding those interactions not related to genes in the dataset, we obtained 121 binary protein-protein interactions. The selected human protein-protein interaction network is shown in Figure S1.

### 2. Inference of gene states via the HMM/GMM hybrid model

The HMM/GMM hybrid model is a classical method in speech recognition [21]. In the present study, we introduced the model to process time series gene expression data. It is known that genome-wide expression profiles contain inherent measurement noise. In order to reduce the effect of this noise, we introduced an HMM/GMM hybrid model into our approach, converting the original gene expression value, which contains noise, into a discrete gene state that represents the qualitative assessment of the gene expression level. Moreover, the HMM/GMM hybrid model takes into account time-dependence, which is a special property of time series data.

A standard continuous HMM is characterized by the following elements [22]: (1) Q, the number of hidden states; (2) A = [a_ij_], the state transition probability distribution, where a_ij_ is the transition probability from state i to j; (3) B = {b_j_(x)}, the emission probability distribution, where b_j_(x) is the emission probability of observing x in state j; and (4) π = {π_i_}, the initial state distribution, where π_i_ is the start point probability of the state i.

An HMM is often simply notated as λ = (A,B,π). The HMM/GMM hybrid model is a specific form of the HMM model, in which the emission probability distribution can be modeled by a mixture of Gaussian functions: 
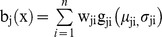
 where n is the mixture number, w_ji_ is the mixture weight, and g_ji_ is the component Gaussian function with expected value μ_ji_ and standard deviation σ_ji_.

We assume that expression values of each gene are from a Markov process, which is widely used by many existing methods [23]. In the model, a state represents the qualitative assessment of gene expression level, reflecting contiguous regions of a time series with similar levels of expression [24]. Each gene remains in one state at a time point. The gene can remain in the same state or switch to other states in the next time point (the number of states is Q). The model is initialized as follows: the initial state distribution (π), state transition probability distribution (A), and mixture weight distribution (w) are set to uniform distribution. The expression values of all genes are divided into Q bins. Each bin has the same number of expression values. The mixture number n is equal to the number of genes. μ_ji_ and σ_ji_ denote the mean and standard deviation of expression values in the jth bin of gene i, respectively. When the initialization process is complete, the model parameter is trained by EM algorithm [25]. Finally, the Viterbi algorithm [26] is applied to infer the hidden state sequences for all genes.

**Algorithm 1:QL_Biclustering Algorithm**

Input: ml, mo, gene state matrix

Output: biclusters

1. **for** each gene expression sequence
2.  Construct all responding suffix strings (SA).
3. **end for**
4. Sort all the suffix strings in SA by MSD (most significant digit) radix sort method.
5. **for** every 2 adjacent suffix strings
6.  Compute longest common prefix and store the longest common prefix length (LCP_Length).
7. **end for**
8. **for** each distinct value i in LCP_Length
9. ** for** each occurrence (j) of i in LCP_Length
10.   pos(i, j) = pinpoint(LCP_Length, i, j)
11.   if i<ml then {Blcp[pos(i,j)] = 1; continue; }
12.   l = max{k| k<pos(i,j) and Blcp[k] = 1}+1
13.   r = min{k| k>pos(i,j) and Blcp[k] = 1}
14.   Blcp[pos(i,j)] = 1
15.   if (r−l+1)<mo then continue;
16.   if >1 && LCP_Length [l -1] =  = i then continue;
17.   Bicluster:
18.    Gene set (G): Gene [l … r];
19.    J(Timepoint (T) and corresponding expression state (S)): SA(pos(i,j), 1...i)
20.  
**end for**
21. **end for**

### 3. Extraction of biclusters through a new biclustering algorithm

Previous approaches of classifying time series expression data have been limited to analysis of genome-wide expression profiles. Gene markers selected by these approaches may be function-related and hence contain redundant information, leading to the degradation of the overall classification performance. Accordingly, it is more effective to regard function-related genes as a whole. We, therefore, extracted biclusters from the gene state sequences of each patient. Biological processes start and finish in a contiguous, but unknown, period, leading to increased activity of sets of genes that can be identified as biclusters with continuous time points. Several authors have previously pointed out the importance of biclusters and their relevance in the identification of biological processes [27], [28].

Biclustering algorithms identify groups of genes that show similar expression patterns under a specific subset of the experimental conditions [29]. Given a gene expression matrix with n genes (rows) and m time points (columns), the temporal biclustering problem is to find a subset of genes G and a continuous segment of time points T, the expression values of genes G follow a desired profile S under time points T, as shown in Figure S2. A bicluster is often simply notated as B(G, J(T, S)). Madeira et al. [30] proposed a suffix tree based CCC-Biclustering method. Suffix tree-based methods are characterized by high space complexity and are difficult for biologists to fully understand. Qu et al. [31] introduced a method based on pairwise alignment of all gene pairs, which can be more easily interpreted, although its time complexity is relatively high.

Here, a new biclustering algorithm, named QL_Biclustering, is proposed to extract biclusters from the gene state matrix obtained in the previous step. In order to differentiate a specific time point, a transformation is introduced, appending a time point to each gene state. For example, given the gene state sequence of a certain gene at each of the first 3 time points S = {3, 2, 1}, the transformed state sequence is J (T, S) = {13, 22, 31}.

QL_Biclustering algorithm (Algorithm 1) is based on the suffix string and longest common prefix. The input is the gene state matrix, ml (minimum number of continuous time points), mo (minimum number of genes). The output is all biclusters satisfying the user's requirements. The algorithm traverses all the values in LCP_Length from the smallest to the largest. For each occurrence of each different value in LCP_Length, QL_Biclustering processes it as follows. At step 10, the jth occurrence position of value i in LCP_Length is pinpointed and stored in pos(i,j). The number of time points of the current bicluster is ensured to be not less than ml at step 11. The algorithm finds an interval [l, r] at steps 12–13, the suffix strings among which share a common prefix with length i. Any bicluster, the gene number of which is less than mo, is ignored at step 15. Step 16 verifies whether the candidate bicluster can be extended to the left. If it does, the candidate bicluster will be discarded because it is not a maximal bicluster.

The QL_Biclustering algorithm is simple but efficient in aspects of both time and space and is linear on the size of the gene state matrix (Supplemental Material S1). In addition, a linear structure is employed to implement our algorithm that, we believe, will be more accessible to biologists.

### 4. Integration of biological network by hypergeometric distribution

In the previous step, we extracted biclusters in which genes showed a similar expression pattern. Genes in a bicluster obtained according to their gene expression values are supposed, but not certain, to be function-related (Supplemental Material S1, Figure S3). Here, we weight a bicluster according to its genes' connection in biological network. In this study, the PPI network is used solely as an illustration: the proposed method is independent of the nature of the network and can be extended to many other biological networks, for example, metabolic networks.

It is known that the distances among genes that are regulated by the same transcription factors in a PPI network are 2 because they have common upstream factors. The distance among genes that belong to a protein complex in a PPI network are 1, as genes represented in a complex are adjacent [31]. Hence, generally, the more closely connected genes in the network, the more likely the genes share a common biological function. In view of this, among all biclusters extracted at a previous step, biclusters in which genes are highly connected relative to overall connectivity in the PPI network are more preferable. Hypergeometric distribution is employed to model the association between gene i and the gene set of bicluster B.



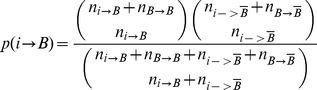
(1)particularly, 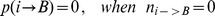 where 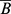 is the set of all genes except genes in bicluster B, n_i->B_ is the number of network interactions between gene i and genes in bicluster B, the remaining notations may be deduced by analogy.

For each bicluster B, its preference (PPIScore) is scored as follows:
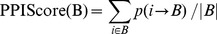
(2)


### 5. Classification of time series gene expression via integration of PPI network

#### 5.1 Computation of similarity between 2 patients based on bicluster similarity and PPIScore

*The* Jaccard Index [30] is used to compute the similarity measure Sim(B_i_,B_j_) between 2 biclusters B_i_(G_i_,J_i_) and B_j_(G_j_,J_j_):
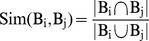
(3)


where |B_i_∩B_j_| = |G_i_∩G_j_|×|J_i_∩J_j_|, |B_i_∪B_j_| = |B_i_|+|B_j_|-|B_i_∩B_j_|, |B_i_| = |G_i_|×|J_i_|, |B_j_| = |G_j_|×|J_j_|.

Given 2 patients, P_1_ and P_2_, firstly, we normalize the PPIScore of all biclusters in P_1_ and P_2_, respectively. Then, the similarity between the 2 patients can be calculated as follows: 
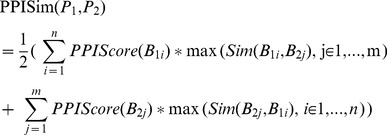
(4)n, m is the number of biclusters in P_1_, P_2_, respectively. B_1i_, B_2j_ denotes the ith bicluster in P_1_, the jth bicluster in P_2_, respectively.

The above formula bears 2 properties, which are crucial to following classifier design:

PPISim(A,A) = 1; (5)

PPISim(A,B) = PPISim(B,A);

#### 5.2 PPI-SVM-KNN classifier

An SVM [32] and KNN [33] hybrid classifier integrating a PPI network named PPI-SVM-KNN (Algorithm 2) is proposed here. The KNN classifier is used at the initial stage, after which an SVM classifier is trained on the collection of K-nearest neighbors. Training an SVM on the entire data set is slow, and the extension of SVM to multiple classes is not as natural as KNN. However, in the neighborhood of a small number of samples, SVM, more often than not, performs better than other classification methods. This is a process with a coarse and quick categorization followed by a finer but slower classification, which inherits the advantages of both SVM and KNN classifiers [34]. In detail, a linear SVM with slack variables was utilized in our approach. PPISim among different patients that satisfies a symmetrical property is used as the kernel matrix (Supplemental Material S1). Because experimental results have shown that nonlinear SVMs, such as the SVM with Gaussian kernel, do not lead to a better performance, we used the simplest linear kernel.

**Algorithm 2: PPI-SVM-KNN**

Input: C, K

Output: predictClass

1. **for** each test patient P do
2.  Calculate the similarity between all patients in the training set and the test patient P and rank those training patients by similarity measure; add the top K patients to kPatients.
3. **if** the label of all the patients in kPatients is class_i
4.   predictClass = class_i;
5. **else**
6.   Compute similarity matrix PPISim among patients in kPatients.
7.   Train linear SVM with slack variable classifier by using PPISim as kernel and predict the label of test patient P.
8.  
**end if**
9. **end for**

The model of linear SVM with slack variables:
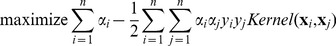
(6)subject to: 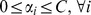; 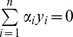


where 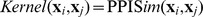



X is the matrix of training samples, y is the vector of corresponding labels, n is the number of samples.

PPISim is calculated as described above.

The label of a new sample P can be predicted as follows: 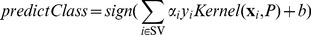
(7)where SV (Support Vector) = {i| α_i_ ≠ 0}


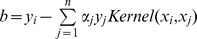
 for any i such that α_i_ ≠ 0.

The parameter C can be viewed as a way to control “softness”: it trades off between maximizing the margin and fitting the training data (minimizing the error). The larger the value of C is, the less likely the classifier is to misclassify samples in the training set. The parameter K specifies the classifier's dependence on the choice of the number of neighbors. When K is small, the algorithm behaves like a straightforward KNN classifier. When K is large enough, our method is totally an SVM. A commonly used strategy to evaluate classification performance is the k-fold cross validation (CV) scheme. In this work, we use 10 repetitions of 4-fold CV, which was also used in previous approaches.

## Results and Discussion

### 1. The HMM/GMM hybrid model is conducive to dealing with time series gene expression

In order to reduce the effect of the measurement noise, we introduced an HMM/GMM hybrid model into our approach, converting the original gene expression value, which contains noise, into a discrete gene state that represents the qualitative assessment of gene expression level. The HMM/GMM hybrid model also takes into consideration time-dependence, which is a special property of time series data. We compared the HMM/GMM hybrid model with many other discretization methods, including Average, Mid-Range, Max-X%Max, Top X%, EFP [35]. The Average All, Average Row, and Average Col discretization methods use the average expression value computed using all the values in the matrix, by row and by column, respectively, to discretize the expression matrix, and the remainder may be deduced by analogy. First, we compared the classification results of distinct discretization methods. The results (Table 1, Table S1) indicate that the HMM/GMM hybrid model yields a better classification performance. Furthermore, we demonstrated the advantages of the GMM/HMM hybrid model in processing time series data from the perspective of class distance. The similarity among all good responders (Pos), among all bad responders (Neg) and between good responders and bad responders (PosNeg) was computed according to Eq.4. D is the ratio of average similarity among patients of the same class and that of different classes. Table S2 indicates that the HMM/GMM hybrid model bears the largest D value among all methods. In other words, when the HMM/GMM hybrid model is employed, the inter-class distances are much larger than intra-class distances, thus leading to better classification results.

**Table 1:** Classification accuracies of different discretization methods for Baranzini dataset and Goertsches dataset: average (AVG) and standard deviation (SD).

Method	AVG/SD	
	Baranzini Dataset	Goertsches Dataset
***HMM/GMM***	***86.20/2.98***	***78.57/8.19***
Average All	79.00/3.81	55.46/7.67
Average Col	76.18/3.26	50.86/8.56
Average Row	81.86/4.85	67.91/8.29
MidRange All	68.39/1.95	55.67/5.61
MidRange Col	65.07/2.89	50.30/9.76
MidRange Row	72.03/3.73	59.55/8.99
Max-X%Max All	63.48/0.02	41.37/2.69
Max-X%Max Col	53.97/5.40	44.32/6.98
Max-X%Max Row	71.34/5.85	58.67/8.72
EFP All	75.16/5.21	61.21/7.47
EFP Col	72.33/3.84	62.10/3.87
EFP Row	78.47/6.04	73.50/5.01
Top X% All	60.57/2.38	54.32/9.26
Top X% Col	52.44/4.96	36.02/7.48
Top X% Row	65.46/3.70	52.51/6.35

### 2. The biclustering *procedure* regards function-related genes as a whole and takes the time course information into consideration as well

**Figure 2:**
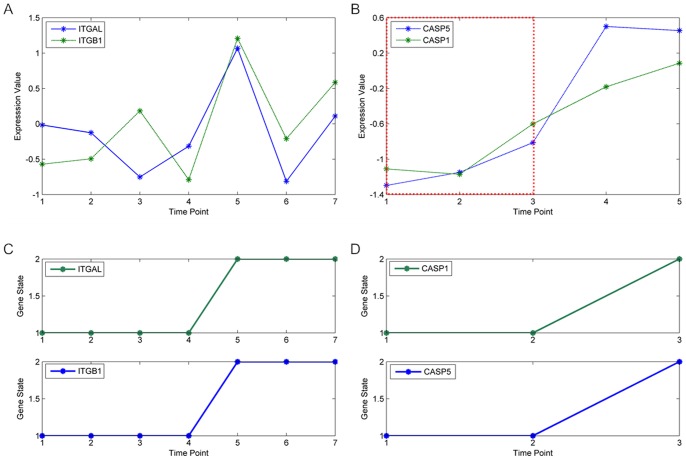
Randomly selected biclustering examples from Baranzini dataset and Goertsches dataset. The expression values of genes in each bicluster are shown in (A) and (B). The state transitions of genes in each bicluster are shown in (C) and (D). The bicluster from Baranzini dataset consists of gene ITGAL and gene ITGB1 and their state transitions from time point 1 to time point 7. The bicluster from Goertsches dataset consists of gene CASP5 and gene CASP1 and their state transitions from time point 1 to time point 3.

Instead of using single gene markers that may be function-related leading to degradation of classification result, the proposed approach regards function-related genes as a whole through biclustering. The biclustering procedure identifies groups of genes that show similar expression patterns under a contiguous segment of time points, which takes the time course information into consideration as well. On average, the number of biclusters inferred from Baranzini dataset is 24 per patient; the number of biclusters inferred from Goertsches dataset is 64 per patient. We randomly selected bicluster examples from each of the two datasets, which are shown in Figure 2. As shown in Figure 2(A), the expression values of gene ITGAL and ITGB1 are consistent from time point 1 to time point 7. Therefore, the state transitions of the two genes are the same, changing from state 1 to state 2 at time point 5 (Figure 2(C)). The biclustering procedure identifies the two genes as a whole from time point 1 to time point 7. In order to check whether the two genes are function-related or not, function enrichment analysis was conducted. As shown in Table S3, ITGAL and ITGB1 share 8 functions (p-value<0.05).The PPIScore of this bicluster from Baranzini dataset is 0.675. As is shown in Figure 2(B), the expression values of gene CASP5 and CASP1 are consistent from time point 1 to time point 3. Hence, the state transitions of the two genes are the same, changing from state 1 to state 2 at time point 3 (Figure 2(D)). These 2 genes share 6 functions (Table S3). The PPIScore of this bicluster from Goertsches dataset is 1. It is worth mentioning that the expression value of gene CASP5 increases significantly from −1 to 0.6 at time point 4. In contrast, the expression value of gene CASP1 increases marginally from −0.6 to −0.2 at time point 4. Therefore, the biclustering procedure only regards the 2 genes as a whole from time point 1 to time point 3.

Many works on static gene expression [8], [36] have reported that multi-gene modules can achieve higher accordance across datasets. For example, Li et al. [36] achieved an average accuracy of 70% across static microarray datasets in prediction of colorectal cancer. Here, we also tested the proposed approach across Baranzini dataset and Goertsches dataset although they are based on different platforms. The two independent datasets were mixed. We randomly selected 75% of the mixed dataset (3 folds) as training set and tested the proposed model on the remaining data (1 fold of the mixed dataset). Ten repetition experiments were executed. Our method achieved an average Accuracy of 77.02%, Precision of 80.12%, Recall of 85.21% and F1Score of 82.15%. These results suggest our prediction through biclustering, which regards function-related genes as a whole, can achieve highly reproducible and acceptable performance across different time series datasets. We also trained the proposed model on Baranzini dataset and tested it on Goertsches dataset, and vice versa. The proposed model achieved an average accuracy of 64.80% and 52.69%, precision of 64.13% and 66.37%, recall of 94.00% and 51.82%, and F1Score of 76.22% and 58.17%. When the model was trained on Goertsches dataset and tested on Baranzini dataset, we found that the average test accuracy is lower than the average training accuracy (which is as high as 80%). One possible reason is that the number of training samples (25) is far less than that of test samples (52). The heterogeneity of the two datasets further aggravates this problem.

### 3. Integration of PPI network into classification of time series gene expression data significantly improves prediction performances

**Figure 3:**
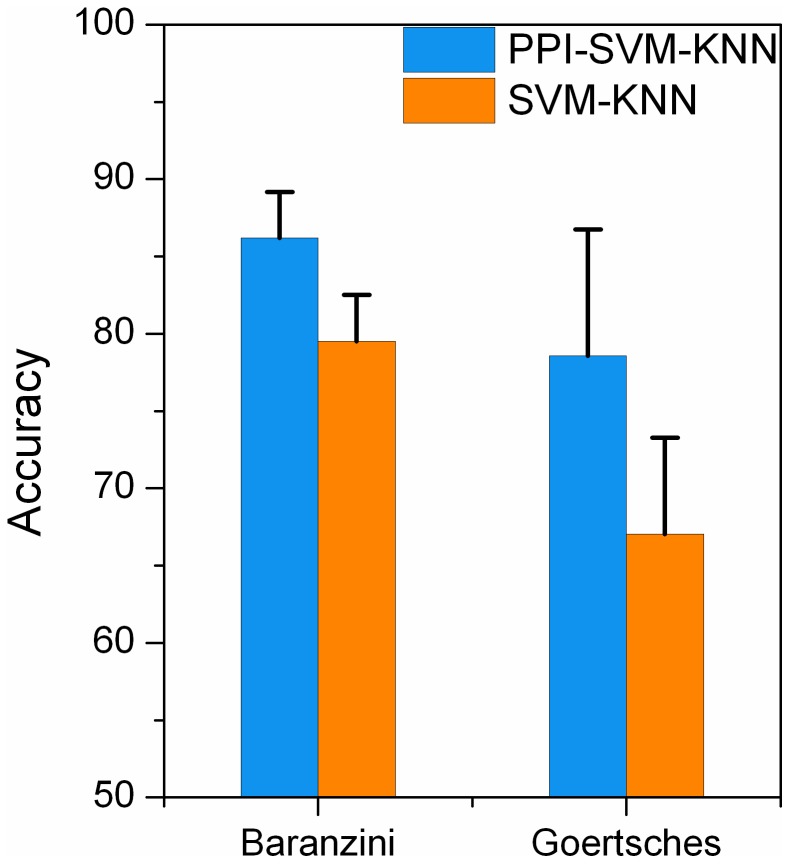
Prediction accuracies of integration (PPI-SVM-KNN) and non-integration (SVM-KNN) of a PPI network. The bars and error ticks represent mean values and standard deviations respectively. The blue bar represents the accuracy of integration PPI network. The orange bar represents the accuracy of non-integration of PPI network.

We next investigated the contribution of integrating PPI network in our method. In the proposed method, we regarded genes that had a similar expression pattern (bicluster) as a whole. Because genes showing similar expression pattern are likely, but not certain to be, function-related, we integrated a PPI network into our approach, weighting a bicluster according to its genes' connection in the network. The more closely connected the genes, the more likely the genes share a common biological function and the higher the weight. We compared the results of integrating a PPI network (PPI-SVM-KNN) to those obtained when no PPI network was integrated (SVM-KNN). As shown in Figure 3 (Table S4), prediction performances are much improved when integrating a PPI network with all other parts unchanged. Integration of PPI network into our approach therefore plays an important role in improving prediction results. Complex diseases arise from the accumulation of both genetic factors and environmental exposition. The environmental signals change the biological information at each level of the hierarchical life system. Data integration approach [37] minimizes the noise that is inherent in data generated through large scale, high-throughput biology. This might explain that integration of PPI network improves prediction performance.

### 4. PPI-SVM-KNN bears clear advantages in classification of time series gene expression

PPI-SVM-KNN employs the HMM/GMM hybrid model to explore the time-dependence property of the data and integrates PPI networks favoring those biclusters that are more likely to share a common biological function, resulting in clear advantages in the classification of time series gene expression. We next demonstrated its advantages in the following aspects: Sensitivity, Accuracy, Precision, Recall and F-measure.

**Figure 4:**
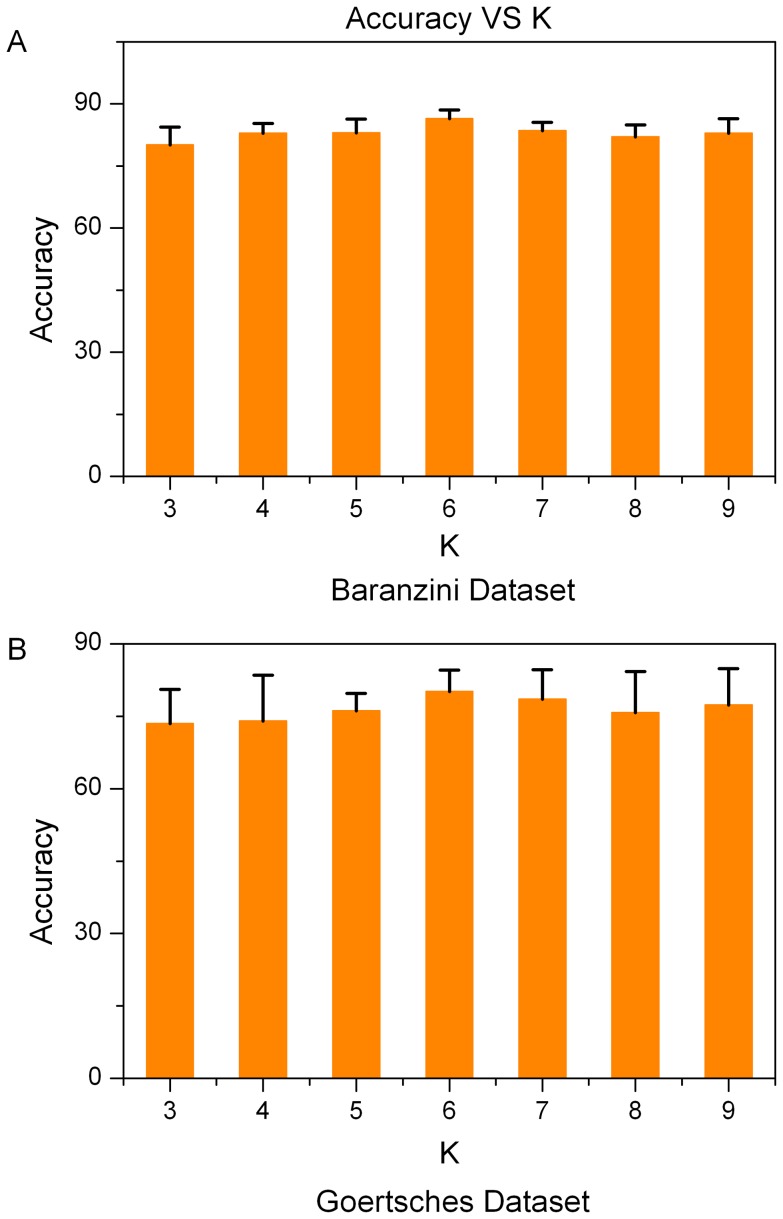
Classification accuracies of PPI-SVM-KNN with the change of parameter K from 3 to 9. The bars and error ticks represent mean values and standard deviations respectively. (A) shows the result for Baranzini dataset. (B) shows the result for Goertsches dataset.

**Figure 5:**
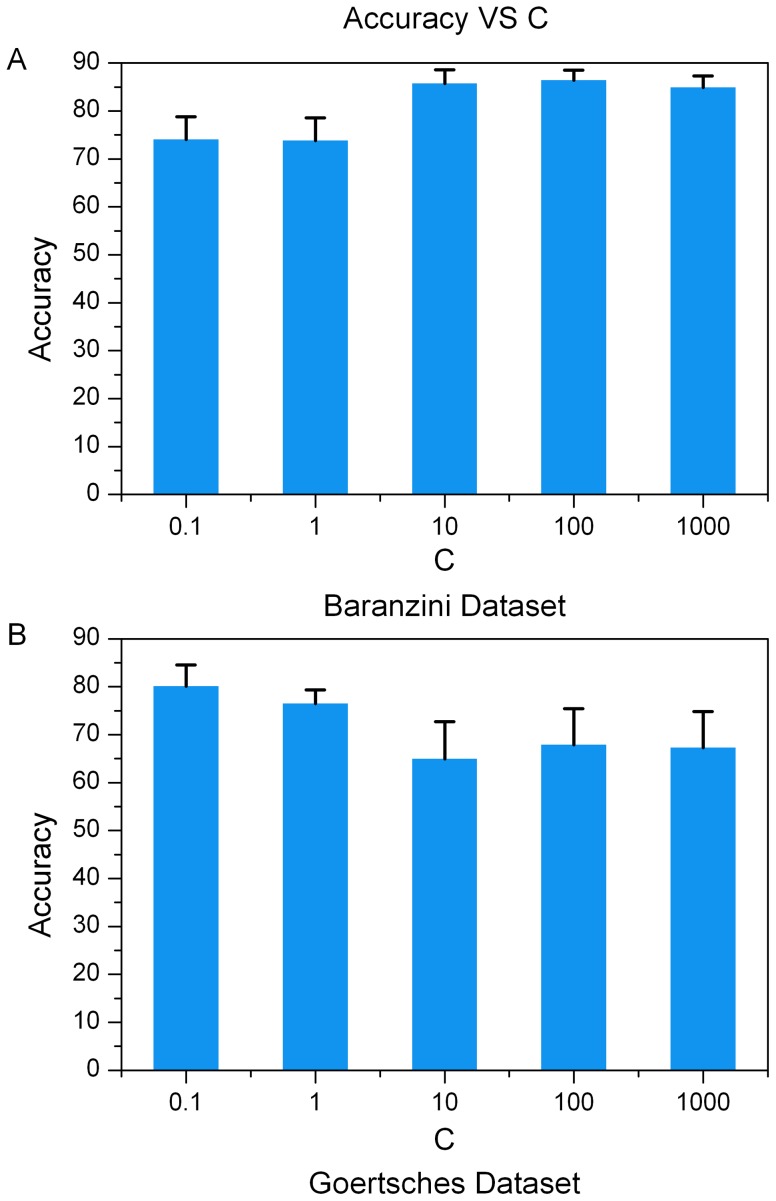
Classification accuracies of PPI-SVM-KNN with the change of parameter C. The bars and error ticks represent mean values and standard deviations respectively. (A) shows the result for Baranzini dataset. (B) shows the result for Goertsches dataset.

We first checked the influence of parameters C and K on classification performances. The parameter C of PPI-SVM-KNN trades off between maximizing the margin and fitting the training data (minimizing the error). The larger the value of C is, the less likely the classifier is to misclassify samples in the training set. The parameter K specifies the classifier's dependence on choice of the number of neighbors. When K is small, the algorithm behaves like a straightforward KNN classifier. When K is large enough, our method is totally an SVM. As shown in Figure 4 and Figure 5 (Table S5 and Table S6), classification results hardly vary and therefore are not very sensitive to the changes of K and C, respectively. In terms of the Baranzini dataset, the accuracy of our approach tends to increase modestly with the growth of C. On the contrary, with regard to Goertsches dataset, the accuracy of our approach tends to decrease marginally with the growth of C. In other words, in order to achieve high accuracy on Goertsches dataset, we need to allow misclassification of some samples in the training set in order to maximize the margin. This, to some extent, indicates that Goertsches dataset might contain noise or that outliers exist in the dataset. Hence, samples in the Goertsches dataset are difficult to classify. Many papers [38], [39], [40] reported that gene expression measurement of a sample between RT-PCR and Affymetrix microarrays often had reduced agreement, especially for those genes with high or low levels of expression. Here, those samples of Baranzini dataset and Goertsches dataset are different. The disagreement between the two dataset might be more obvious. This might be a reason for the different transition of accuracy on the two datasets with the growth of parameter C. It is worth mentioning that, in all other experiments of this work, the parameter C and K are selected by cross validation.

Table 2 demonstrates the classification accuracy of distinct methods in 2 different MS patient response datasets. We compared our method with several state-of-the-art algorithms [12], [17], [41], [42], [43], [44], as well as with standard classifiers SVM and random forest (The results of other common classifiers on Baranzini dataset have been reported in the work of Carreiro et al. [42]). The dataset used in standard classifiers consists of a single matrix where each row represents the data for a single patient. The results of all those methods listed here are based on 70 genes (Baranzini dataset) or 58 genes (Goertsches dataset) without any further feature selection, with the exception of the IBIS method, which used only the first time point expression data and 3 selected genes. The source code of IBIS is not available and the HMMConst3 source code does not work; hence, we listed only their original results on the Baranzini dataset. For other approaches that were available, we evaluated them on both the Baranzini and Goertsches datasets. As shown in Table 2, PPI-SVM-KNN outperformed previous methods that are based solely on gene expression profiles with regard to accuracy. As for Goertsches dataset that is noisier than the Baranzini dataset and consists of fewer samples, the classification accuracy of the dsSVM, HMMClass, SVM methods is markedly low. However, PPI-SVM-KNN still achieved good classification performance on the noisier and smaller Goertsches dataset. Hence, compared with previous approaches, our method achieved more stability in prediction accuracy across 2 different datasets.

**Table 2:** Classification accuracies of distinct classification methods for Baranzini dataset and Goertsches dataset: average (AVG) and standard deviation (SD).

Method	AVG/SD	Reference
Baranzini dataset		
IBIS	74.20	Baranzini, et al., 2004
dsSVM	73.44/2.56	Borgwardt, et al., 2006
Meta-Profiles	59.42/6.17	Carreiro, et al., 2011
HMMConst3	81.38/10.00	Costa, et al., 2009
HMMClass	78.42/3.44	Lin, et al., 2008
uHONMFtf	60.97/2.91	Li and Ngom, 2011
Random forest	70.19/5.39	
SVM	67.47/1.90	
***PPI-SVM-KNN***	***86.20/2.98***	
**Goertsches dataset**		
dsSVM	55.75/3.28	Borgwardt, et al., 2006
Meta-Profiles	64.00/4.62	Carreiro, et al., 2011
uHONMFtf	53.37/6.94	Li and Ngom, 2011
HMMClass	45.82/4.30	Lin, et al., 2008
Random forest	52.39/3.24	
SVM	57.26/4.21	
***PPI-SVM-KNN***	***78.57/8.19***	

**Figure 6:**
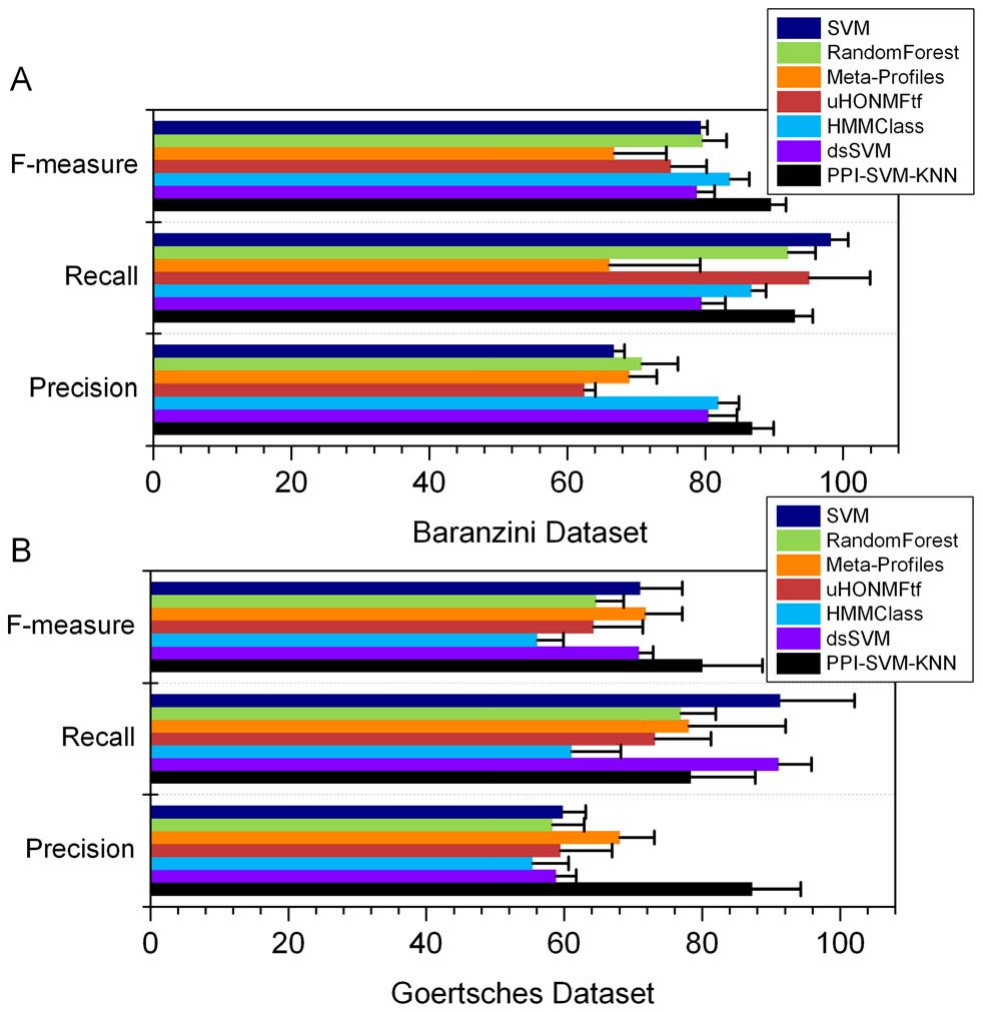
Precision, Recall and F-measure of different classification approaches. The bars and error ticks represent mean values and standard deviations respectively. (A) shows the result for Baranzini dataset. (B) shows the result for Goertsches dataset.

Because the prediction accuracy *per se* is insufficient to comprehensively measure the quality of a classifier, other commonly used performance criteria such as Recall, Precision, and F-measure [45] were tested. We noticed, however, that only prediction accuracy was reported in the papers of the state-of-the-art methods. Recall is the ratio of correctly detected good responders to all patients that actually are good responders. Precision is the ratio of correctly predicted good responders to all predicted good responders. F-measure is the harmonic mean of precision and recall. As shown in Figure 6, our approach performed well with regard to all of the above metrics, and outperformed other methods in terms of F-measure and Precision. The Recall value of our approach on Goertsches dataset is slightly lower than that of dsSVM and SVM. In other words, compared with dsSVM and SVM, the prediction of a truly good responder as a bad responder would more likely happen in our approach. In general, the approach we report here is an improvement on other previous methods based on overall consideration of Accuracy, Precision, Recall, and F-measure.

### 5. PPI-SVM-KNN achieved better prediction results on early-stage data

**Figure 7:**
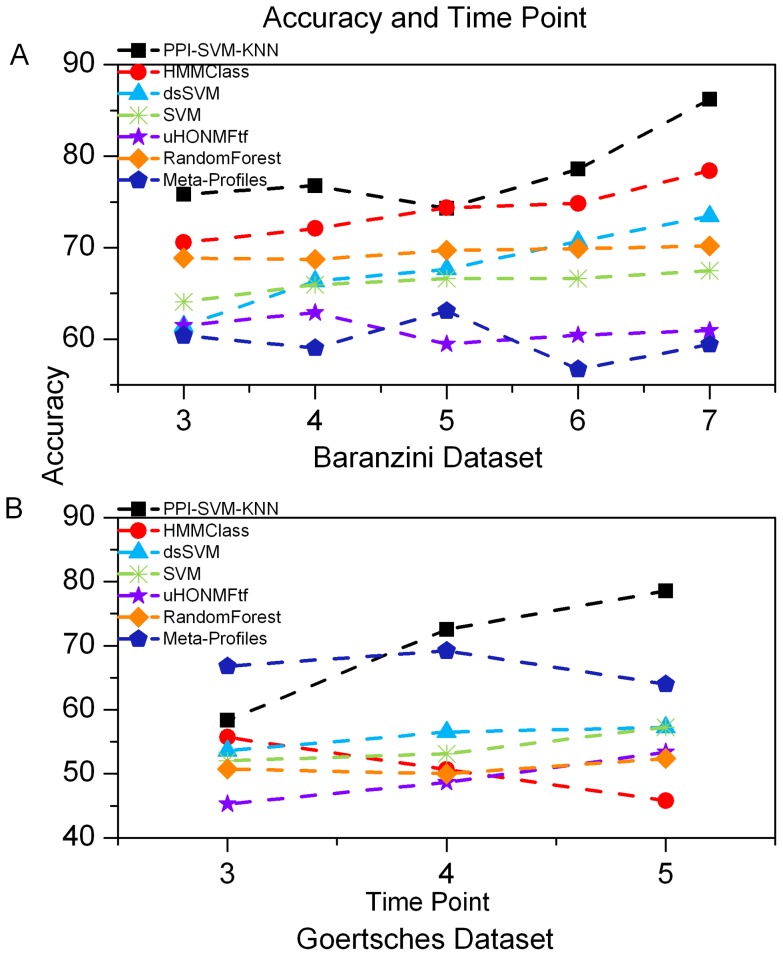
Prediction accuracies of different classification approaches with the change of measurements. The points in the figure represent mean values. (A) shows the accuracies from time point 3 to time point 7 for Baranzini dataset. (B) shows the accuracies from time point 3 to time point 5 for Goertsches dataset.

Considering that accurate prediction from early-stage data is of great significance in clinical diagnosis, we evaluated the influence of the number of measurements on classification performances and compared it with other methods. We repeated our classification experiment considering the first n measurements (for all n ≥ 3). As expected, classification performances increase as the number of measurements grows (Figure 7, Table S7). In comparison with other methods, our approach almost outperformed them at all values of n. We emphasize that our method achieved good results even on early-stage data (n = 3, 4), implying the potential of our approach in practical prediction. After patients have been treated with rIFN-β for a short period, for example, (n = 3), our approach can predict their probable responses, offering suggestions to those patients as to whether they should continue to receive the drug therapy or not.

### 6. Discussion and future work

Personalized medicine, the use of marker-assisted diagnosis and targeted therapies derived from individual's molecular profile, is involving in the pharmaceutical industry and medical practice and it is likely to affect many aspects of society[46]. Currently, many works have been proposed to predict individual's therapy response based on static gene expression [47], [48], including the work of Ruiz-Peña et al. [49], which has been put into clinical practice. However, the accuracy of those approaches is, at best, at the 80 percent level. Further improvements are required before these methods can be widely used in clinical application. The main challenges in personalized medicine are as follows. Firstly, gene signatures have been developed using a number of different platforms and with samples coming from different clinical series [50]. Secondly, in clinical studies, samples are often limited. Noise is inherent in data generated through large-scale, high throughput experiments. Finally, complex diseases arise from the accumulation of both genetic factors and environmental exposition. The environmental signals change the biological information at each level of the hierarchical system. Therefore, understanding of complex disease requires the integration of different data types arising from DNA, RNA, protein, metabolites, small molecules and many different aspects of phenotype [37], [51].

Here, we developed a novel method to predict the MS patients' response to IFN-Beta, which is a work toward personalized medicine. The proposed approach is based on the idea of integrating two hierarchies of life system-gene and protein. For this specific MS prediction problem, we did not design a feature selection component. The 70 genes were selected by Baranzini [17] on the basis of their presumed biological action. The feature set includes genes coding for type I and II IFN-responsive molecules, cytokine receptors, members of the interferon (IFN) signaling and apoptosis pathways, and several transcriptions factors involve in immune regulation. In a word, the author selected the genes that are closely related to MS and the medicine (IFN-Beta). It is worth pointing out that the feature selection component is needed when the proposed method is applied to other prediction problems. Feature selection can be performed either by the similar method mentioned in the work of Baranzini, or by some other widely used methods in clinical studies [2], such as “N” t test statistics [52], cluster index scores [53] and the modified t test statistics [54]. In addition, in order to achieve better prediction on the patients' therapy response, we are striving to integrate metabolic profiles into our model since metabolomics is considered the one that comes closest to expressing phenotype [55].

## Conclusion

In this paper, we presented a sound and reliable methodology for the classification of clinical time series data based on the novel idea of integrating biological networks. Admittedly, there are few points that we would like to improve in the near future. For example, during the process of data discretization (HMM/GMM hybrid model), time course information of the data is, more or less, lost. However, our method outperformed prior approaches that do not consider biological networks with regard to various performance criteria. Compared with other approaches, our method achieved more stability in prediction across 2 different datasets. Moreover, the proposed method achieved high accordance in prognosis prediction across independent time series datasets. Finally, we found that our approach achieved better prediction performances on early-stage data, implying that our method has great potential in clinical prediction. All the results on the 2 independent datasets have indicated that integration of the network into classification of time series significantly improves prediction performance, which is similar to that of static gene expression demonstrated by recent research groups [8].

## Supporting Information

Figure S1 **The selected binary protein protein interaction network.** Each node represents a gene. Each edge represents a protein protein interaction, i.e. there exists a interaction between the two proteins which are encoded by the two genes the edge connects.
(PDF)

Figure S2 **Biclustering process.** Biclusters were extracted from (A) the gene state matrix. In order to differentiate specific time point, a transformation is introduced, appending time point to each gene state (B). Biclusters extracted from gene state matrix are shown in (C).
(PDF)

Figure S3 **Functional enrichment analysis of genes in each Bicluster.** The function of genes in each bicluster was analyzed. (A) is the result of Baranzini Dataset; (B) is the result of Goertsches Dataset. In (A) and (B), the horizontal axis represents the range of function count; the left vertical axis represents the number of biclusters; the right vertical axis represents the cumulative frequency. The height of each bar represents the number of biclusters. The x-axis label of each bar represents function counts (e.g. the left most bar of (A) indicates that there are nearly 30% biclusters associating with 1∼50 functions). The line represents cumulative frequency of corresponding bar in the Figures.
(PDF)

Table S1 **Precision, Recall and F-measure of different discretization methods on Baranzini dataset and Goertsches dataset: average (AVG) and standard deviation (SD).**
(PDF)

Table S2 **Patient similarity of different discretization methods on Baranzini dataset and Goertsches dataset.**
(PDF)

Table S3 **Function enrichment analysis of the bicluster examples on Baranzini dataset and Goertsches dataset.** Gene functions with p-value <0.05 are selected here.
(PDF)

Table S4 **Precision, Recall and F-measure of integration versus non-integration of PPI network on Baranzini dataset and Goertsches dataset: average (AVG) and standard deviation (SD).**
(PDF)

Table S5 **Precision, Recall and F-measure of PPI-SVM-KNN with the change of K from 3 to 9: average (AVG) and standard deviation (SD).**
(PDF)

Table S6 **Precision, Recall and F-measure of PPI-SVM-KNN with the change of C from 0.1 to 1000: average (AVG) and standard deviation (SD).**
(PDF)

Table S7 **Precision, Recall and F-measure of distinct approaches with the change of measurements: average (AVG) and standard deviation (SD).**
(PDF)

Supplemental Material S1 **1. Complexity analysis of the proposed biclustering algorithm and its comparison with CCC-Biclustering; 2. Function enrichment analysis of bicluster; 3. Kernel validation.**
(PDF)
