# Supplementary figures and images for: Classification of Time Series Gene Expression in Clinical Studies via Integration of Biological Network
Source: PLoS One. 2013 Mar 13;8(3):e58383. doi: 10.1371/journal.pone.0058383 (PMC3596388; doi:10.1371/journal.pone.0058383)

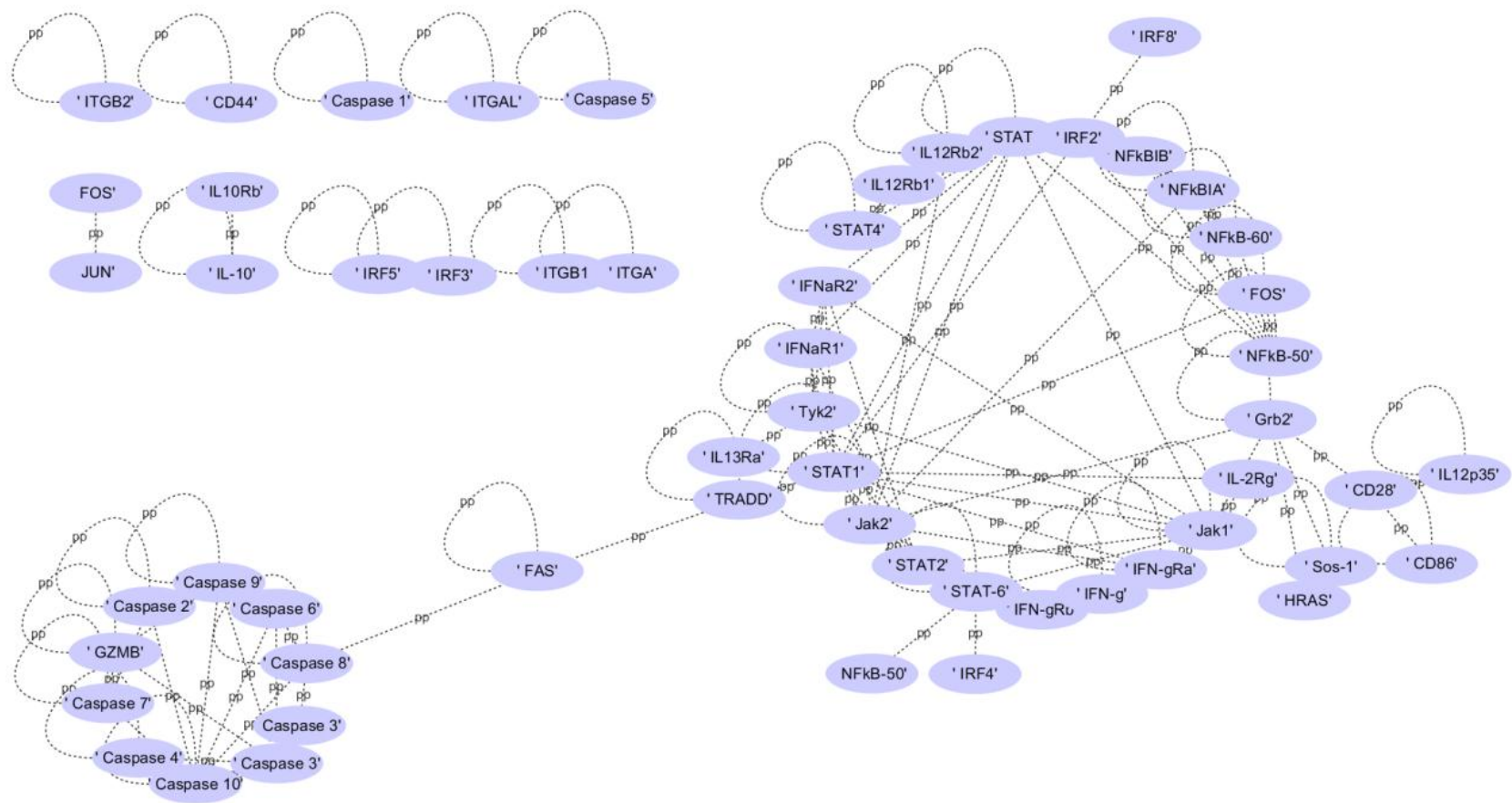

Supplement: Figure S1 — The selected binary protein protein interaction network. Each node represents a gene. Each edge represents a protein protein interaction, i.e. there exists a interaction between the two proteins which are encoded by the two genes the edge connects. (PDF) [file pone.0058383.s001.pdf]

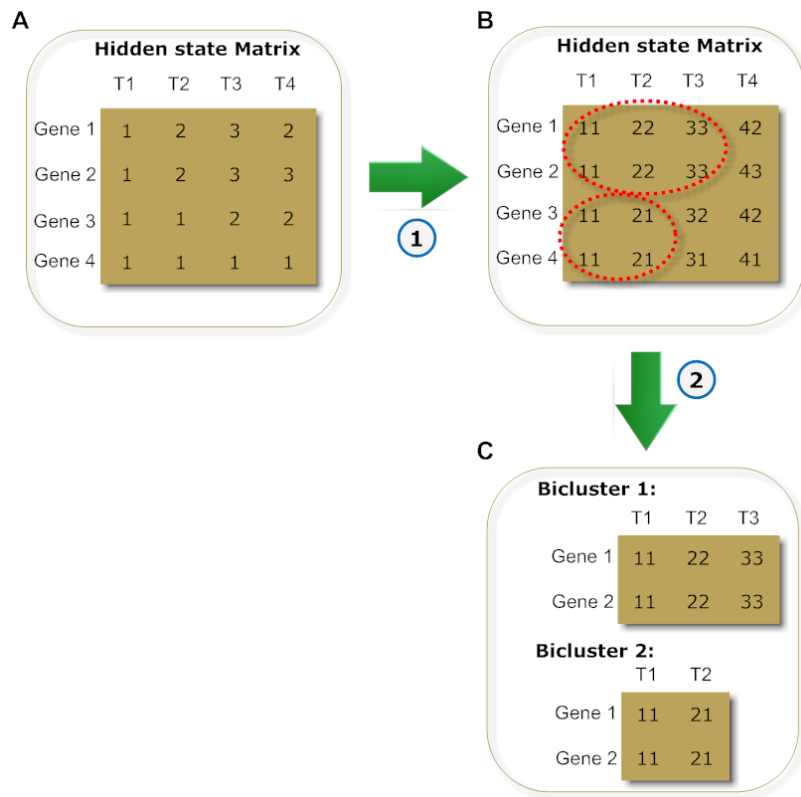

Supplement: Figure S2 — Biclustering process. Biclusters were extracted from (A) the gene state matrix. In order to differentiate specific time point, a transformation is introduced, appending time point to each gene state (B). Biclusters extracted from gene state matrix are shown in (C). (PDF) [file pone.0058383.s002.pdf]

A

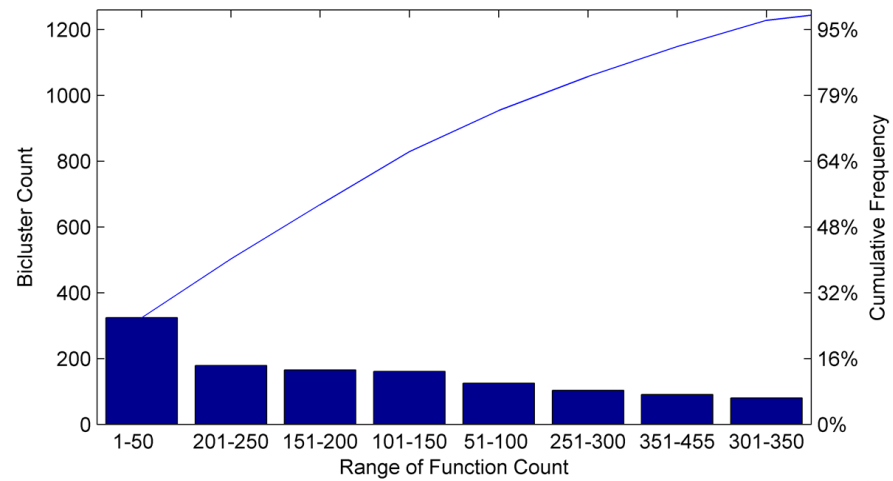

Baranzini Dataset

B

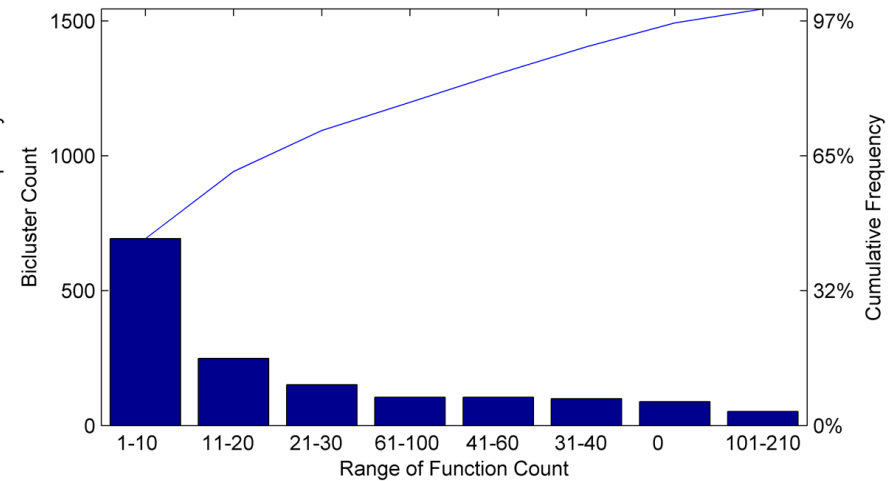

Goertsches Dataset

Supplement: Figure S3 — Functional enrichment analysis of genes in each Bicluster. The function of genes in each bicluster was analyzed. (A) is the result of Baranzini Dataset; (B) is the result of Goertsches Dataset. In (A) and (B), the horizontal axis represents the range of function count; the left vertical axis represents the number of biclusters; the right vertical axis represents the cumulative frequency. The height of each bar represents the number of biclusters. The x-axis label of each bar represents function counts (e.g. the left most bar of (A) indicates that there are nearly 30% biclusters associating with 1∼50 functions). The line represents cumulative frequency of corresponding bar in the Figures. (PDF) [file pone.0058383.s003.pdf]
