# Supplementary material for: Classification of Time Series Gene Expression in Clinical Studies via Integration of Biological Network
Source: PLoS One. 2013 Mar 13;8(3):e58383. doi: 10.1371/journal.pone.0058383 (PMC3596388; doi:10.1371/journal.pone.0058383)
Supplement: Table S1 — Precision, Recall and F-measure of different discretization methods on Baranzini dataset and Goertsches dataset: average (AVG) and standard deviation (SD). (PDF) [file pone.0058383.s004.pdf]

Table S1. Precision, Recall and F-measure of different discretization methods on Baranzini dataset and Goertsches dataset: average (AVG) and standard deviation (SD)

A) Baranzini Dataset

**Method**	**Precision**	**Recall**	**F-measure**
GMM/HMM	***86.80/3.13***	***92.98/2.61***	***89.49/2.25***
Average All	81.88/3.54	88.02/3.10	84.17/2.64
Average Col	79.00/2.43	87.22/3.26	782.40/2.47
Average Row	84.17/4.18	89.13/4.93	86.04/3. 78
MidRange All	68.01/1.48	95.79/1.46	79.39/1.07
MidRange Col	65.22/1.87	97.25/3.95	77.89/2.07
MidRange Row	71.78/3.54	95.13/1.57	81.43/2.33
Max - X%Max All	63.48/0.02	1/0	77.62/0.03
Max - X%Max Col	63.58/4.32	64.13/8.30	63.01/5.84
Max - X%Max Row	72.84/4.30	88.88/7.62	79.49/4.71
EFP All	78.16/3.20	84.72/6.46	80.52/4.97
EFP Col	77.69/3.87	80.27/3.71	78.32/2.99
EFP Row	81.70/4.46	87.60/5.48	83.83/4.50
Top X% All	70.86/2.86	64.96/3.04	66.85/2.10
Top X% Col	60.87/3.86	69.58/4.23	64.37/3.79
Top X% Row	79.84/4.62	62.56/3.79	68.89/3.36

B) Goertsches Dataset

**Method**	**Precision**	**Recall**	**F1Score**
GMM/HMM	***87.22/7.06***	***78.33/9.37***	***80.02/8.73***
Average All	63.52/8.11	66.04/9.26	62.90/6.12
Average Col	62.13/10.59	61.04/7.08	58.30/6.49
Average Row	78.25/10.61	66.87/9.98	NaN
MidRange All	65.22/5.72	65.20/9.77	61.81/6.47
MidRange Col	55.12/11.85	63.33/10.35	NaN
MidRange Row	68.00/8.49	63.12/10.62	63.97/9.07
Max - X%Max All	NaN	6.46/11.80	NaN
Max - X%Max Col	NaN	46.88/9.73	NaN
Max - X%Max Row	62.41/6.59	81.87/9.57	69.72/7.13
EFP All	68.01/10.41	67.50/6.53	NaN
EFP Col	73.88/4.51	63.75/7.74	64.58/3.50
EFP Row	84.67/8.43	71.04/7.37	74.55/4.63
Top X% All	62.30/11.45	57.50/6.45	NaN
Top X% Col	42.53/7.92	52.08/11.94	NaN
Top X% Row	58.13/5.96	74.16/5.90	63.86/5.02

NaN denotes that there exist, at least, one time both the numerator and the denominator equal zero among ten repetitions of 4 fold CV. Taking Precision for example. There exist , at least, one time both True Positive and False Positive equal zero among ten repetitions of 4 fold CV.
