# Supplementary material for: Classification of Time Series Gene Expression in Clinical Studies via Integration of Biological Network
Source: PLoS One. 2013 Mar 13;8(3):e58383. doi: 10.1371/journal.pone.0058383 (PMC3596388; doi:10.1371/journal.pone.0058383)
Supplement: Table S2 — Patient similarity of different discretization methods on Baranzini dataset and Goertsches dataset. (PDF) [file pone.0058383.s005.pdf]

Table S2. Patient similarity of different discretization methods on Baranzini dataset and Goertsches dataset.

A) Baranzini Dataset

**Discretization**	**Pos**	**Neg**	**PosNeg**	**Normalized D**
GMM/HMM	***0.2252***	***0.2180***	***0.1938***	***1***
Average All	0.1460	0.1420	0.1380	0.4816
Average Col	0.1459	0.1419	0.1378	0.4847
Average Row	0.1470	0.1466	0.1412	0.4302
MidRange All	0.9698	0.9471	0.9579	0.2562
MidRange Col	0.3498	0.2589	0.2927	0.8276
MidRange Row	0.1751	0.1662	0.1638	0.5051
Max - X%Max All	1	0.9995	0.9998	0.2234
Max - X%Max Col	0.7827	0.7474	0.7610	0.3095
Max - X%Max Row	0.6070	0.5670	0.5843	0.3352
EFP All	0.1386	0.1415	0.1353	0.3743
EFP Col	0.1368	0.1395	0.1338	0.3621
EFP Row	0.1468	0.1469	0.1420	0.3949
Top X% All	0.5127	0.6127	0.5577	0.0359
Top X% Col	0.4922	0.5914	0.5402	0
Top X% Row	0.4110	0.4253	0.4132	0.2393

B) Goertsches Dataset

**Method**	**Pos**	**Neg**	**PosNeg**	**Normalized D**
GMM/HMM	***0.0879***	***0.0923***	***0.0849***	***1***
Average All	0.1499	0.1519	0.1510	0.1996
Average Col	0.1499	0.1519	0.1510	0.1996
Average Row	0.1494	0.1550	0.1490	0.4548
MidRange All	0.2028	0.2220	0.2102	0.1382
MidRange Col	0.1869	0.1936	0.1879	0.3272
MidRange Row	0.1511	0.1590	0.1521	0.3862
Max - X%Max All	0.9822	0.9975	0.9893	0.2144
Max - X%Max Col	0.6527	0.6843	0.6737	0
Max - X%Max Row	0.4800	0.5095	0.4954	0.0548
EFP All	0.1520	0.1528	0.1506	0.4110
EFP Col	0.1516	0.1557	0.1497	0.5583
EFP Row	0.1527	0.1558	0.1501	0.5987
Top X% All	0.5412	0.5492	0.5464	0.1774
Top X% Col	0.5333	0.5243	0.5369	0.0787
Top X% Row	0.4749	0.4418	0.4604	0.3990

Pos represents the average similarity among patients who are good responders to Drug therapy. **Neg** represents the average similarity among patients who are bad responders. **PosNeg** represents the average similarity between good responders and bad responders. **D** is the ratio of average similarity among patients of the same response and that of different responses. Normalized D is computed by adjusting the D value of all the methods to the range of [0, 1]. In detail, normalized D of value x (x in D) is computed as follows: normalized D (x) = $\frac{x - \min(D)}{\max(\mathrm{D}) - \min(D)}$.
