# Supplementary material for: Classification of Time Series Gene Expression in Clinical Studies via Integration of Biological Network
Source: PLoS One. 2013 Mar 13;8(3):e58383. doi: 10.1371/journal.pone.0058383 (PMC3596388; doi:10.1371/journal.pone.0058383)
Supplement: Table S3 — Function enrichment analysis of the bicluster examples on Baranzini dataset and Goertsches dataset. Gene functions with p-value <0.05 are selected here. (PDF) [file pone.0058383.s006.pdf]

Table S3. Function enrichment analysis of the bicluster examples on Baranzini dataset and Goertsches dataset. Gene functions with p-value < 0.05 are selected here.

**NO.**	**P-value**	**Function ID**	**Function Name**
**Bicluster Example of Baranzini Dataset**			
1	2.69E-03	GO:0007159	leukocyte cell-cell adhesion
2	2.08E-02	GO:0045785	positive regulation of cell adhesion
3	7.66E-03	GO:0007229	integrin-mediated signaling pathway
4	3.16E-02	GO:0043235	receptor complex
5	1.16E-03	GO:0008305	integrin complex
6	1.66E-02	KEGG:04810	Regulation of actin cytoskeleton
7	2.17E-02	KEGG:04514	Cell adhesion molecules (CAMs)
8	5.35E-03	KEGG:04670	Leukocyte transendothelial migration
**Bicluster Example of Goertsches Dataset**			
1	2.42E-02	GO:0071214	cellular response to abiotic stimulus
2	2.35E-02	GO:0009612	response to mechanical stimulus
3	3.56E-03	GO:0071260	cellular response to mechanical stimulus
4	3.08E-02	GO:0008234	cysteine-type peptidase activity
5	6.53E-03	GO:0004197	cysteine-type endopeptidase activity
6	1.48E-03	KEGG:04621	NOD-like receptor signaling pathway
