# Supplementary material for: Classification of Time Series Gene Expression in Clinical Studies via Integration of Biological Network
Source: PLoS One. 2013 Mar 13;8(3):e58383. doi: 10.1371/journal.pone.0058383 (PMC3596388; doi:10.1371/journal.pone.0058383)
Supplement: Table S4 — Precision, Recall and F-measure of integration versus non-integration of PPI network on Baranzini dataset and Goertsches dataset: average (AVG) and standard deviation (SD). (PDF) [file pone.0058383.s007.pdf]

Table S4. Precision, Recall and F-measure of integration versus non-integration of PPI network on Baranzini dataset and Goertsches dataset: average (AVG) and standard deviation (SD)

**Method**	**Precision**	**Recall**	**F-measure**
**Baranzini Dataset**			
PPI-SVM-KNN	***86.80/3.13***	***92.98/2.61***	***89.49/2.25***
SVM-KNN	79.50/3.01	83.14/2.63	86.63/3.43
**Goertsches Dataset**			
PPI-SVM-KNN	***87.22/7.06***	***78.33/9.37***	***80.02/8.73***
SVM-KNN	NaN	61.04/7.55	NaN
