# Supplementary material for: Classification of Time Series Gene Expression in Clinical Studies via Integration of Biological Network
Source: PLoS One. 2013 Mar 13;8(3):e58383. doi: 10.1371/journal.pone.0058383 (PMC3596388; doi:10.1371/journal.pone.0058383)
Supplement: Table S5 — Precision, Recall and F-measure of PPI-SVM-KNN with the change of K from 3 to 9: average (AVG) and standard deviation (SD). (PDF) [file pone.0058383.s008.pdf]

Table S5. Precision, Recall and F-measure of PPI-SVM-KNN with the change of K from 3 to 9: average (AVG) and standard deviation (SD)

Method	Precision	Recall	F-measure
**Baranzini Dataset**			
3	83.55/2.72	86.94/4.01	84.59/3.52
4	84.66/2.20	90.03/2.55	86.92/1.69
5	84.98/2.98	89.83/2.99	87.04/2.90
6	86.15/2.10	94.48/1.28	89.82/1.45
7	83.53/2.08	93.13/1.51	87.76/1.46
8	82.07/2.21	93.33/3.79	86.95/2.14
9	82.70/3.56	93.65/2.24	87.53/2.50
**Goertsches Dataset**			
3	83.17/8.18	73.54/8.04	76.43/6.54
4	82.00/11.56	74.37/8.90	NaN
5	87.38/7.53	75.21/6.98	78.24/2.69
6	89.96/5.37	76.88/6.01	81.10/4.67
7	87.29/5.72	77.29/9.08	80.36/6.69
8	86.29/8.79	75.00/5.38	77.59/6.29
9	85.58/6.72	76.88/8.07	79.60/6.73
