# Supplementary material for: Classification of Time Series Gene Expression in Clinical Studies via Integration of Biological Network
Source: PLoS One. 2013 Mar 13;8(3):e58383. doi: 10.1371/journal.pone.0058383 (PMC3596388; doi:10.1371/journal.pone.0058383)
Supplement: Table S6 — Precision, Recall and F-measure of PPI-SVM-KNN with the change of C from 0.1 to 1000: average (AVG) and standard deviation (SD). (PDF) [file pone.0058383.s009.pdf]

Table S6. Precision, Recall and F-measure of PPI-SVM-KNN with the change of C from 0.1 to 1000: average (AVG) and standard deviation (SD)

C	Precision	Recall	F-measure
**Baranzini Dataset**			
0.1	83.88/4.68	74.37/5.09	77.29/4.31
1	83.68/4.69	74.37/5.09	77.18/4.23
10	85.84/2.33	94.24/3.17	89.40/2.49
100	86.42/2.06	86.15/2.10	94.48/1.28
1000	85.43/2.28	93.61/3.10	88.73/2.04
**Goertsches Dataset**			
0.1	89.96/5.37	76.88/6.01	81.10/4.67
1	87.79/4.62	75.21/4.44	78.04/3.35
10	69.26/8.17	78.75/8.26	NaN
100	72.89/6.11	78.96/8.01	73.94/6.43
1000	70.21/7.65	82.50/4.30	74.64/5.29
