# Supplementary material for: Classification of Time Series Gene Expression in Clinical Studies via Integration of Biological Network
Source: PLoS One. 2013 Mar 13;8(3):e58383. doi: 10.1371/journal.pone.0058383 (PMC3596388; doi:10.1371/journal.pone.0058383)
Supplement: Table S7 — Precision, Recall and F-measure of distinct approaches with the change of measurements: average (AVG) and standard deviation (SD). (PDF) [file pone.0058383.s010.pdf]

Table S7. Precision, Recall and F-measure of distinct approaches with the change of measurements: average (AVG) and standard deviation (SD).

A) Precision

Time Point	PPI-SVM-KNN	HMMClass	dsSVM	SVM	uHONMFtf	Meta-Profiles	Random Forest
**Baranzini Dataset**							
3	79.64/2.57	77.18/3.95	66.39/3.50	64.80/2.11	63.60/2.17	67.15/4.07	70.61/3.23
4	79.64/3.48	77.98/2.88	69.70/3.51	65.78/1.52	64.46/1.24	71.11/6.22	69.70/1.93
5	75.88/3.00	79.45/4.03	72.84/4.13	66.26/1.06	62.92/2.38	67.21/4.05	70.31/1.71
6	79.88/4.06	78.93/4.51	77.44/3.40	66.26/1.36	63.78/5.14	69.83/5.25	71.07/3.00
7	86.80/3.13	81.88/3.03	80.40/4.17	66.72/1.55	62.42/1.62	68.99/4.00	70.77/5.29
**Goertsches Dataset**							
3	65.01/8.20	NaN	56.99/3.39	56.07/3.04	53.63/6.39	68.28/5.51	57.26/4.20
4	79.63/6.17	57.08/4.71	58.57/1.92	56.96/2.05	56.15/5.97	72.13/4.16	56.53/2.74
5	87.22/7.06	55.36/5.26	59.21/2.84	59.76/3.35	59.40/7.51	68.02/5.01	58.27/4.61

B) Recall

Time Point	PPI-SVM-KNN	HMMClass	dsSVM	SVM	uHONMFtf	Meta-Profiles	Random Forest
**Baranzini Dataset**							
3	84.97/3.74	78.13/4.34	80.56/6.00	95.87/3.92	91.18/7.96	73.03/11.99	89.38/5.23
4	86.81/3.24	80.00/4.83	83.82/5.47	97.57/2.12	93.68/6.56	60.61/12.04	91.08/3.42
5	89.41/3.06	81.91/7.28	81.67/5.01	97.85/3.44	89.06/6.56	82.73/5.35	92.26/3.91
6	90.38/4.65	84.38/4.40	78.30/5.96	97.99/2.29	90.14/9.64	56.06/14.79	90.21/4.49
7	92.98/2.61	96.67/2.16	79.44/3.50	98.19/2.56	95.07/8.82	66.06/13.23	91.98/3.98
**Goertsches Dataset**							
3	72.50/8.26	67.71/9.41	87.08/7.90	86.25/8.35	59.79/7.19	86.67/13.33	75.62/5.38
4	75.00/9.21	72.29/7.35	91.87/7.86	88.54/8.32	64.58/8.20	80.67/9.14	76.67/7.01
5	78.33/9.37	61.04/7.18	92.50/7.57	91.25/10.82	73.12/8.13	78.00/14.07	76.88/5.06

C) F-measure

Time Point	PPI-SVM-KNN	HMMClass	dsSVM	SVM	uHONMFtf	Meta-Profiles	Random Forest
**Baranzini Dataset**							
3	81.52/2.43	76.65/1.45	72.09/3.92	77.11/1.59	74.39/4.18	69.64/7.52	78.34/2.98
4	82.55/2.71	78.36/3.41	75.43/4.13	78.45/1.40	75.90/2.65	64.76/7.61	78.61/2.00
5	81.55/2.67	79.86/5.27	75.98/3.26	78.83/1.65	73.10/2.53	74.00/2.76	79.45/3.02
6	84.33/3.35	80.65/3.84	76.75/4.27	78.90/1.27	73.39/5.69	61.24/10.45	79.00/3.43
7	89.49/2.25	83.57/2.82	78.75/2.58	79.34/0.99	75.02/5.16	66.80/7.58	79.60/3.50
**Goertsches Dataset**							
3	66.80/6.60	NaN	68.12/4.69	66.91/5.92	NaN	75.50/3.51	63.28/4.78
4	75.01/6.85	62.75/5.06	71.08/3.09	68.62/2.57	NaN	75.73/2.74	63.72/4.93
5	80.02/8.73	56.08/3.77	71.73/3.92	70.95/6.18	64.18/7.22	71.81/5.27	64.60/4.01
