# Supplementary material for: Classification of Time Series Gene Expression in Clinical Studies via Integration of Biological Network
Source: PLoS One. 2013 Mar 13;8(3):e58383. doi: 10.1371/journal.pone.0058383 (PMC3596388; doi:10.1371/journal.pone.0058383)
Supplement: Supplemental Material S1 — 1. Complexity analysis of the proposed biclustering algorithm and its comparison with CCC-Biclustering; 2. Function enrichment analysis of bicluster; 3. Kernel validation. (PDF) [file pone.0058383.s011.pdf]

Supplemental Material for "*Classification of Time Series Gene Expression in Clinical Studies via Integration of Biological Network*"

Liwei Qian[1], Haoran Zheng[1,2,3,*], Hong Zhou[1], Ruibin Qin[1] and Jinlong Li[1]

[1] School of Computer Science and Technology, University of Science and Technology of China, Hefei, People's Republic of China.

[2] Anhui Key Laboratory of Software Engineering in Computing and Communication, University of Science and Technology of China, Hefei, People's Republic of China.

[3]Department of Systems Biology, University of Science and Technology of China, Hefei, People's Republic of China.

*To whom correspondence should be addressed. Email: hrzheng@ustc.edu.cn

1. Complexity analysis of the proposed biclustering algorithm and its comparison with CCC-Biclustering

Let the input matrix be an R row by C column matrix. Let the symbols of the input matrix is from an alphabet ∑ (with|∑|symbols). The algorithm 1 mainly consists of 4 parts. In order to construct suffix strings, the algorithm traverses the input matrix once. Therefore, the time complexity (steps 1-3) is O(RC). At step 4, the algorithm sorts all the suffix strings with time complexity of O(RC^2). At steps 5-7, the algorithm traverses the suffix array once with time complexity of O(RC) since the length of the suffix array is RC. The last part of the algorithm (steps 8-21) traverses the LCP_length once with time complexity of O(RC) since the length of the LCP_Length is RC. Hence, the time complexity of the algorithm is O(RC^2). As the largest space in the algorithm is the space of the input matrix, the space complexity of QL-Biclustering algorithm is O(RC). In comparison, the time and space complexity of CCC-Biclustering are O(RC) and O(RC^2|∑|) [1].

2. Function enrichment analysis of bicluster

We hypothesized that most genes in the same bicluster might share a common biological function since these genes show similar expression pattern. In order to validate this hypothesis, we used g:Profiler [2], a tool for functional characterization of genes, to analyze the function of genes in a bicluster. We extracted biclusters from expressions of all patients. After excluding redundant biclusters, genes in every bicluster are analyzed through g:Profiler. As shown in Supplementary Figure S3, most biclusters (97.54% biclusters in Baranzini dataset and 94.24% biclusters in Goertsches dataset) share some functions.

3. Kernel validation

The model of linear SVM with slack variables:

$$\text{maximize } \sum_{i=1}^{n}\alpha_i - \frac{1}{2}\sum_{i=1}^{n}\sum_{j=1}^{n}\alpha_i\alpha_j y_i y_j Kernel(\mathbf{x}_i,\mathbf{x}_j) \tag{1}$$

subject to: $0 \le \alpha_i \le C, \forall i$; $\sum_{i=1}^{n}\alpha_i y_i = 0$

where

$$Kernel(\mathbf{x}_i,\mathbf{x}_j) = \text{PPIS}im(\mathbf{x}_i,\mathbf{x}_j)$$

X is the matrix of training samples and y is the vector of corresponding labels.

To ensure that a kernel function actually corresponds to some feature space, it must be symmetric, i.e., Kernel(x_i,x_j) = Kernel(x_j,x_i). As we mentioned in the manuscript (section 2.5.1), the PPISim kernel satisfies this property. Typically, kernels are also required to satisfy Mercer's theorem, which states that the kernel matrix K should be positive semi-definite, i.e., it has non-negative eigenvalues[3]. This condition ensures that the solution of the formula (1) produces a global optimum. However, good results have been achieved with non-Mercer kernels, and convergence is expected when the SMO algorithm is used, despite no guarantee of optimality when non-Mercer kernels are used [4-8]. Furthermore, despite its wide use, the Sigmoid kernel matrix is not positive semi-definite for certain values of the parameters γ and θ[9]. In short, indefinite kernel can still work and achieve good classification results. The proposed kernel PPISim is positive semi-definite in most cases. Although the semi-definite property might not be guaranteed, our results have showed that it still works well and has achieved good classification results.

REFERENCES

1. Ukkonen E (1995) On-line construction of suffix trees. Algorithmica 14: 249-260.
2. Reimand J, Kull M, Peterson H, Hansen J, Vilo J (2007) g: Profiler—a web-based toolset for functional profiling of gene lists from large-scale experiments. Nucleic acids research 35: W193-W200.
3. Cristianini N, Shawe-Taylor J (2000) An introduction to support vector machines and other kernel-based learning methods: Cambridge university press.
4. Howley T, Madden MG (2005) The genetic kernel support vector machine: Description and evaluation. Artificial Intelligence Review 24: 379-395.
5. Bahlmann C, Haasdonk B, Burkhardt H (2002) Online handwriting recognition with support vector machines-a kernel approach. Frontiers in Handwriting Recognition, Eighth International Workshop on: IEEE. pp. 49-54.
6. Chen J, Ye J (2008) Training SVM with indefinite kernels. Proceedings of the 25th international conference on Machine learning: ACM. pp. 136-143.
7. Haasdonk B (2005) Feature space interpretation of SVMs with indefinite kernels. Pattern Analysis and Machine Intelligence, IEEE Transactions on 27: 482-492.
8. Wu G, Chang EY, Zhang Z (2005) An analysis of transformation on non-positive semidefinite similarity matrix for kernel machines. Proceedings of the 22nd International Conference on Machine Learning: Citeseer.
9. Lin HT, Lin CJ (2003) A study on sigmoid kernels for SVM and the training of non-PSD kernels by SMO-type methods. Neural Computation: 1-32.
